# Why Ophthalmology: A Cross-Sectional Study of the Motivating Factors Influencing the Choice of Ophthalmology as a Career Among Medical Students in Saudi Arabia

**DOI:** 10.7759/cureus.20844

**Published:** 2021-12-31

**Authors:** Ma’an A Al-Barry, Turki Fehaid Algethami, Ahmed Marshoud Alsaedi, Rayan Nasser Alahmadi, Alwaleed Khalid Bardisi, Mohammed Saad Khoshhal, Abdulaziz Saud Alharbi

**Affiliations:** 1 Ophthalmology, College of Medicine, Taibah University, Al-Madinah Al-Munawwarah, SAU; 2 Medicine, College of Medicine, Taibah University, Al-Madinah Al-Munawwarah, SAU; 3 College of Medicine, Taibah University, Al-Madinah Al-Munawwarah, SAU

**Keywords:** future career, career, medical student, future specialty, ophthalmology

## Abstract

Background: Choosing a future career is a frightening and anxious decision for medical students; understanding any factor that influences making such a decision will be helpful for the medical students to reach it with satisfaction.

Aim: The aim of this study was to determine the factors that would influence the choice of ophthalmology as a future career among medical students in multi-medical colleges in Saudi Arabia.

Methods: This cross-sectional study was conducted for pre-clinical and clinical medical students from February 2021 to May 2021 in multi-medical colleges in Saudi Arabia. The questionnaire used was from previously published research, and it was modified and validated by a board-certified ophthalmologist.

Results: A total of 6.1% of all medical students considered ophthalmology as their first choice as a future career; factors that influenced choosing ophthalmology were personal interest, satisfaction from improving patient quality of vision, good patient prognosis, and the appeal of being an ophthalmologist; these factors showed a highly significant difference in comparison to other factors.

Conclusion: Several factors are explored for choosing ophthalmology as a future specialty; understanding these factors can help medical students determine their choices for a future career.

## Introduction

One of the most important decisions a medical student has to make is choosing a future specialty that perfectly matches his/her abilities and preferences. Medical students usually face this dilemma during or before their internship [1]. Multiple factors would influence the choice of medical specialties, which can be classified into two main domains: (1) personal-related such as gender, type of personality, and preferences and (2) work-related such as the environment of the specialty, specialty income, and affection on lifestyle and the workload [2-6]. In the United States, the most influencing factor considered when choosing a medical specialty as a future career was the perception of the others toward the job, followed by having more days off work [7].

According to a previous study done in Jordan, the most significant factors influencing the preference of future specialty among medical students were "intellectual content of the specialty," "individual’s competencies," and "reputation of the specialty" [3]. Locally, a study done in Al-Madinah Al-Munawwarah concluded that the most important predictors for the selection of medical career were personality preferences and specialty characters [8]. Another study done in Riyadh revealed that the choice of the future medical specialty depends mainly on "anticipated income," "specialty interest," and "specialty flexibility" [9].

Ophthalmology is an interesting specialty that gives the opportunity of using surgical skills and the knowledge to diagnose and treat with medicine. The choice of the Saudi residents who were matched into the ophthalmology residency program was made depending mainly on the ability to combine surgery and medicine [10]. In Saudi Arabia, ophthalmology is considered a highly competitive specialty due to the limited number of seats. Therefore, knowing the factors that push medical students toward ophthalmology is critical to understanding the challenges that prevent graduates from choosing this specialty and thus directing the efforts to solve these challenges.

Looking at the literature locally, a study on medical students at King Saud bin Abdulaziz University for Health Sciences in Riyadh revealed that the most important factors for the students to consider ophthalmology as a future career were high income, private sector opportunities, and part-time opportunities, respectively [11]. Although there is a study done before exploring why medical students prefer ophthalmology as a future career in one university, there are rare studies on locally addressing this issue across all regions in Saudi Arabia at many universities. Therefore, the authors conducted this study to determine factors that would influence the choice of ophthalmology as a future career among medical students at different universities in Saudi Arabia. This study might lead to a better understanding of why ophthalmology is a highly competitive field among graduated medical students. In addition, it is interesting to consider which factors contribute the most to choosing ophthalmology as a future specialty during college years among medical graduates.

## Materials and methods

A cross-sectional study was conducted in Saudi Arabia between February 2021 to May 2021; a structured and self-administrated electronic questionnaire was distributed among medical students of multiple health faculties in Saudi Arabia, and data were collected using an online platform. Arabic version of the questionnaire that was used in previously published research was used; it was modified and validated by a board-certified ophthalmologist [11]. Male and female medical students in both pre-clinical and clinical phases, with the majority of participants in the clinical phase, were included in the study. The exclusion criteria in our study comprised of those who did not accept the consent or graduated medical students and interns.

The questionnaire was divided into three sections: The first section was about the demographic features of the study subjects. While the second section mainly focused on influential factors that led to choosing or rejecting ophthalmology as a future specialty. The third section was concentrated on academic performance and opportunities.

The total number of participants was 465, with more than 65% of studied subjects who were active in the clinical phase in the period between February 2021 and May 2021. 

In our study, we used Statistical Package for Social Sciences (SPSS) program (version 27.0, IBM corp. Armonk, NY, USA) for data analysis and management. Results were expressed as categorical data using frequencies and percentages for each variable. Chi-square was used to compare these data. All tests were two-sided. The significance level was expressed as highly significant, significant, and non-significant according to the P-value, P < 0.001, P < 0.05, P > 0.05, respectively.

The agreement of individuals to fill the questionnaire was considered as a consent of participation. The data are highly confidential, and it is only accessible by the primary investigator and co-investigators. All of them participated voluntarily, and they were able to withdraw from the study. In addition, the data did not include any identification. The ethical approval for this study was received from the Scientific Research Ethics Committee of College of Medicine at Taibah University, Al-Madinah Al-Munawwarah, Saudi Arabia (STU-20-023).

## Results

Table 1 shows some basic information about the sample population, which included the total number of 865 participants that was almost divided by half between the two genders (413 males and 452 females). Of those, 831 (95.7%) were single, 27 (3.1%) were married, and seven (0.8%) were divorced. The average age of participants was 23.5 years, with the youngest age of 20 years. Most of our participants were in their fourth year of school with a number of 276 (31.8%), and the highest percentage of students who chose ophthalmology was in the fifth year accounting for 11.7%. Regarding the grade point average (GPA) of our participants, the highest recorded GPA (26.4%) was above 4.75 out of 5, which correlated with the choice of ophthalmology as a future career as it recorded the highest number among other GPAs; 34.7% of our participants made their choice of specialty during their clinical years, and 15.7 made it during their basic years, while only 8.9% made it before medical school, and the rest (40.4%) chose other.

**Table 1:** Characteristics information of participants and their specialty preferences GPA, Grade point average.

	Total		Ophthalmology		Surgical		Medical		Others		P-value
	n	%	n	%	n	%	n	%	n	%	
Gender											
Male	413	47.6	29	7	123	29.8	136	32.9	125	30.3	0.048
Female	452	52.1	24	5.3	102	22.6	168	37.2	158	35	
Marital status											
Divorced	7	0.8	0	0	4	57.1	2	28.6	1	4.3	0.223
Married	27	3.1	2	7.4	8	29.8	13	48.1	4	14.8	
Single	831	95.7	51	6.1	213	25.6	289	34.8	278	33.5	
Age											
20	157	18.1	9	5.7	45	28.7	30	19.1	73	46.5	<0.0001
21	156	18	3	1.9	32	20.5	60	38.5	61	39.1	
22	227	26.2	17	7.5	52	22.9	84	37	74	32.6	
23	182	21	18	9.9	44	24.2	71	39	49	26.9	
24	87	10	6	6.9	28	32.2	35	40.2	18	20.7	
25	29	3.3	0	0	13	44.8	11	37.9	5	17.2	
26	23	2.6	0	0	10	43.5	10	43.5	3	13	
>26	4	0.5	0	0	1	25	3	75	0	0	
Level											
2^nd ^year	157	18.1	9	5.7	47	29.9	32	20.4	69	43.9	<0.0001
3^rd^ year	121	13.9	4	3.3	29	24	40	33.1	48	39.7	
4^th^ year	276	31.8	11	4	64	23.2	108	39.1	93	33.7	
5^th^ year	180	20.7	21	11.7	46	25.6	59	32.8	54	30	
6^th^ year	131	15.1	8	6.1	39	29.8	65	49.6	19	14.5	
GPA											
<3.5	56	6.5	3	5.4	12	21.4	23	41.1	18	32.1	0.016
3.5–3.75	127	14.6	5	3.9	29	22.8	61	48	32	25.2	
3.75–4	119	13.7	7	5.9	27	22.7	47	39.5	38	31.9	
4–4.25	77	8.9	3	3.9	22	28.6	25	32.5	27	35.1	
4.25–4.5	123	14.2	3	2.4	32	26	46	37.4	42	34.1	
4.5–4.75	134	15.4	12	9	36	26.9	48	35.8	38	28.4	
>4.75	229	26.4	20	8.7	67	29.3	54	23.6	88	38.4	
When did you make specialty preference?											
Before medical school	77	8.9	8	10.4	28	36.4	35	45.5	6	7.8	0.001
During basic science	136	15.7	15	11	54	39.7	66	48.5	1	0.7	
During clinical years	301	34.7	19	6.3	99	32.9	151	50.2	32	10.6	
Others	351	40.4	11	3.1	44	12.5	52	14.8	244	69.5	

**Figure 1:**
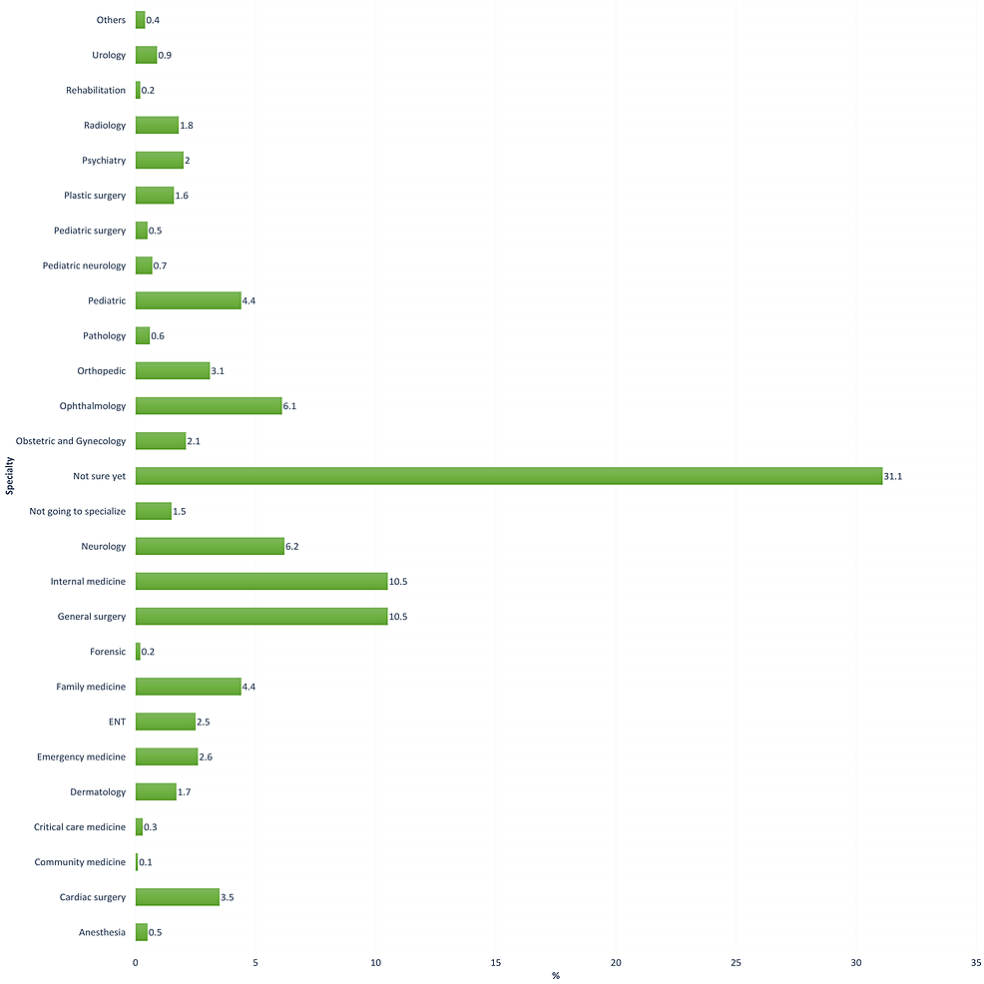
Specialties of interest among participants

Figure 1 shows the preferred specialties of our participants, where only 53 (6.1%) of those chose ophthalmology as their preferred future specialty, while 61.3% chose other medical and surgical specialties. Others were either not sure yet (31.1%) or were not going to specialize (1.5%).

**Figure 2:**
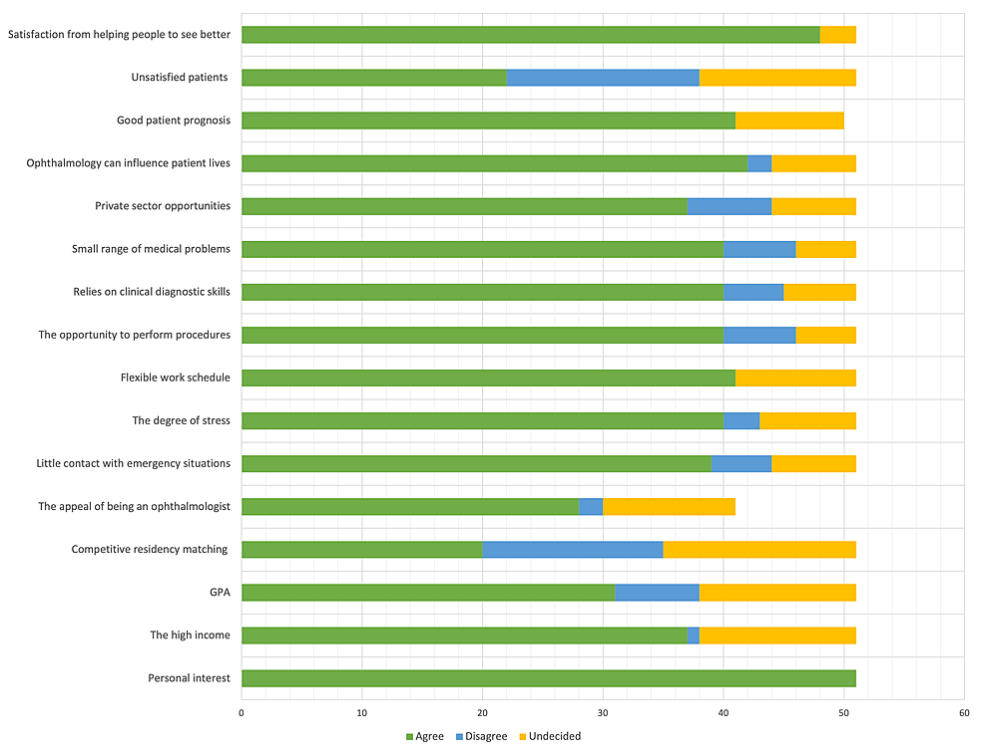
Factors that attracted the study participants toward ophthalmology and other factors that pushed them away

Figure 2 shows the factors that attracted our participants toward ophthalmology and other factors that pushed them away, respectively.

Table 2 explains the significant difference regarding the factors that influence the choice of ophthalmology as a career. Personal factors like personal interest (P < 0.001) and the appeal of being an ophthalmologist (P < 0.0001) showed a significant difference, while others like the high income (P = 0.081) and GPA (P = 0.281) showed an insignificant difference. The work style of ophthalmology and its effect on the lifestyle showed a significant difference in influencing medical students to choose this specialty. These factors included having a little contact with emergency situations (P = 0.005), degree of stress (P = 0.023), flexible work schedule (P = 0.005), and private sector opportunities (P = 0.001). The clinical aspect of ophthalmology showed a significant difference, for example, how it relies on clinical diagnostic skills (P = 0.011), the opportunity to perform procedures (P = 0.007), and the small range of medical problems (P = 0.036). Factors that would demonstrate long-term effects like how ophthalmologists can influence patients’ lives (P = 0.001), the high possibility of good patient prognosis (P = 0.003), and the personal satisfaction from helping people to see better (P = 0.004) showed significant difference. However, having unsatisfied patients (P = 0.226) showed an insignificant difference. The difficulty of getting into ophthalmology residency (P = 0.054) showed an insignificant difference in choosing ophthalmology as a future career.

**Table 2:** Factors that influence the choice of ophthalmology as a career GPA, Grade point average.

Question	Answer	Specialty no. (%)		P-value
		Ophthalmology	Others	
Personal interest	Agree	12 (4.6)	251 (95.4)	<0.001
	Disagree	0	64 (100)	
	Strongly agree	39 (13)	261 (87)	
	Strongly disagree	0	48 (100)	
	Undecided	0	138 (100)	
High income	Agree	20 (5.6)	337 (94.4)	0.081
	Disagree	1 (1.6)	61 (98.4)	
	Strongly agree	17 (10.1)	151 (89.9)	
	Strongly disagree	0	32 (100)	
	Undecided	13 (6.8)	179 (93.2)	
Your GPA	Agree	16 (6.2)	241 (93.8)	0.281
	Disagree	7 (6.4)	103 (93.6)	
	Strongly agree	15 (8.2)	168 (91.8)	
	Strongly disagree	0	63 (100)	
	Undecided	13 (6.7)	182 (93.3)	
The appeal of being ophthalmologist	Agree	20 (7.8)	236 (92.2)	<0.0001
	Disagree	2 (2)	96 (98)	
	Strongly agree	18 (13.4)	116 (86.6)	
	Strongly disagree	0	60 (100)	
	Undecided	11 (4.4)	237 (95.6)	
The desire for little contact with emergency situations	Agree	19 (6.3)	282 (93.7)	0.005
	Disagree	4 (4.1)	93 (95.9)	
	Strongly agree	20 (12.6)	139 (87.4)	
	Strongly disagree	1 (2.1)	47 (97.9)	
	Undecided	7 (3.7)	184 (96.3)	
The degree of stress	Agree	22 (7.9)	257 (92.1)	0.023
	Disagree	3 (3.7)	79 (96.3)	
	Strongly agree	18 (10.1)	161 (89.9)	
	Strongly disagree	0	31 (100)	
	Undecided	8 (3.6)	213 (96.4)	
Lifestyle/flexible work schedule	Agree	14 (4.4)	306 (95.6)	0.005
	Disagree	0	38 (100)	
	Strongly agree	27 (10.9)	221 (89.1)	
	Strongly disagree	0	22 (100)	
	Undecided	10 (6.1)	812 (93.9)	
Private sector opportunities	Agree	15 (4.8)	297 (95.2)	0.001
	Disagree	7 (15.6)	38 (84.4)	
	Strongly agree	22 (10.5)	187 (89.5)	
	Strongly disagree	0	31 (100)	
	Undecided	7 (3.6)	186 (96.4)	
The opportunity to perform procedures	Agree	21 (6.9)	282 (93.1)	0.007
	Disagree	6 (12.8)	41 (87.2)	
	Strongly agree	19 (9.4)	184 (90.6)	
	Strongly disagree	0	30 (100)	
	Undecided	5 (2.5)	196 (97.5)	
Relies on clinical diagnostic skills	Agree	22 (7.3)	278 (92.7)	0.011
	Disagree	5 (9.4)	48 (90.6)	
	Strongly agree	18 (10)	162 (90)	
	Strongly disagree	0	30 (100)	
	Undecided	6 (2.6)	222 (97.4)	
Routine work/small range of medical problems	Agree	24 (7.9)	281 (92.1)	0.036
	Disagree	6 (9.4)	58 (90.6)	
	Strongly agree	16 (8.1)	182 (91.9)	
	Strongly disagree	0	39 (100)	
	Undecided	5 (6.1)	812 (93.9)	
The difficulty of getting into ophthalmology residency	Agree	12 (4.9)	232 (95.1)	0.054
	Disagree	12 (13.2)	79 (86.8)	
	Strongly agree	8 (4.7)	164 (95.3)	
	Strongly disagree	3 (5.4)	53 (94.6)	
	Undecided	16 (7)	214 (93)	
The likelihood that an ophthalmologist can influence patients’ lives	Agree	17 (6)	267 (94)	0.001
	Disagree	1 (1.5)	66 (98.5)	
	Strongly agree	25 (12.3)	178 (87.7)	
	Strongly disagree	1 (3.4)	28 (96.6)	
	Undecided	7 (3.4)	201 (96.6)	
Good patient prognosis	Agree	15 (5)	286 (95)	0.003
	Disagree	0	39 (100)	
	Strongly agree	26 (11.3)	212 (88.7)	
	Strongly disagree	0	28 (100)	
	Undecided	9 (5)	172 (95)	
Unsatisfied patients	Agree	16 (8)	184 (92)	0.226
	Disagree	13 (9.4)	126 (90.6)	
	Strongly agree	6 (6.3)	90 (93.8)	
	Strongly disagree	3 (4.8)	59 (95.2)	
	Undecided	13 (4.4)	280 (95.6)	
Personal satisfaction from helping people to see better	Agree	27 (8.8)	280 (91.2)	0.004
	Disagree	0	42 (100)	
	Strongly agree	21 (8.4)	228 (91.6)	
	Strongly disagree	0	34 (100)	
	Undecided	3 (1.9)	159 (98.1)	

Upon asking participants if they have ever been exposed to ophthalmology, 66.2% were exposed in different ways and times. On the other hand, 33.6% were never exposed to ophthalmology (Table 3).

**Table 3:** Exposure to ophthalmology

Exposure to ophthalmology	Frequency	Percent (%)
Yes, in clinical and basic years	272	31.4
Yes, in basic years	305	35.3
Neither	288	33.3

Table 4 includes some questions that were answered by yes or no by our participants. The first question was “Are you interested in ophthalmology as a future career?”: 44.4% of participants answered “yes,” and 55.3% were not interested. Another question was “Is ophthalmology on top of your list for a future career?”: 28% of participants answered “yes” to this question, while 71.7% said “no.” The next question was “Have you ever been exposed to an ophthalmic procedure?”: 28.5% said “yes,” while 71.2% answered “no.” The last question was “Have you ever observed an ophthalmic procedure?”: 32.4% answered “yes,” while 67.3% said “no.”

**Table 4:** Answers of participants on their interest in ophthalmology

Questions		Frequency	Percent (%)	P-value
Have you ever been exposed to an ophthalmic procedure?	Yes	247	28.5	0.002
	No	618	71.2	
Have you ever observed an ophthalmic procedure?	Yes	281	32.4	<0.0001
	No	584	67.3	

Table 5 includes the questions to assess the participants’ interest in different ophthalmology activities. Upon asking them if they have ever attended an ophthalmology conference, only 7.6% answered “yes,” whereas 30.6% did not attend but were interested in attending in the future. On the other hand, 46.8% and 14.6% answered “no” and “not my priority,” respectively. When asking participants, “Have you ever participated in an ophthalmology research?”, 7.9% said “yes,” and 17.2% were not involved in research at the moment but showed interest. However, 66.2% did not participate, and 8.3% were never interested in participating. Finally, when they were asked, “Have you ever participated in any community service activity related to ophthalmology?”, 10% said they have participated in at least one community service, whereas 15.2% did not participate but showed interest. On the other hand, 68.1% had no experience, and 6.3% had no plans to participate.

**Table 5:** Answers of participants on their interest in ophthalmology activities

Questions		Frequency	Percent (%)
Have you ever attended a conference in ophthalmology?	Yes	66	7.6
	No	406	46.8
	I would like to	266	30.6
	Not my priority	127	14.6
Have you ever participated in ophthalmology research?	Yes	69	7.9
	No	575	66.2
	I would like to	149	17.2
	Not my priority	72	8.3
Have you ever participated in any community service activity related to ophthalmology?	Yes	87	10
	No	591	68.1
	I would like to	132	15.2
	Not my priority	55	6.3

## Discussion

Choosing a future career has been always a dilemma for medical students. The process consumes a long time and is affected by many influences. Students need to review what are the advantages and disadvantages of each medical field, explore the options for their professional and academic advancement, cope with the specialty with their lifestyle preferences, try their favorable specialty in their elective courses, and consider the income and benefit. However, certain specialties have developed a reputation for being a high demand over time; one of these top specialties is ophthalmology, which has become harder to get in because of the limited seats in the training facilities and the competitiveness of this specialty [2,3,12,13].

The purpose of this research was to investigate the causes and factors that influence medical students on their decision to choose ophthalmology as a future career.

The study was done on 865 medical students, which showed that general surgery and internal medicine were the highest preferred specialties (10.5%) followed by neurology (6.2%) and ophthalmology (6.1%). However, most students (31.1%) have not chosen their preferred specialty yet. These findings match what was previously published in King Saud bin Abdulaziz University for Health Sciences, except that ophthalmology was the second most preferable specialty and pediatric was the third [11].

Surprisingly, personal interest was the highest influencing factor in choosing ophthalmology as a future career, whereas 96% of the study participants chose it as their most important determining factor. This came opposing to two research published in King Saud bin Abdulaziz University for Health Sciences and Alfaisal University, which showed that high income was the most significant factor influencing the choice of ophthalmology as a future career [9,11].

Vision is considered one of the most important senses in human lives, so losing this sense could present a significant effect on patients. Therefore, 91% of participants chose personal satisfaction from helping people to see better as their second most important factor in choosing ophthalmology. Notably, good patient prognosis came in third with 79% giving it credit that goes together with personal satisfaction from helping people to see better. Also, influencing patients’ lives was one of the most significant factors, with as much as 79% for choosing ophthalmology as a future career. This aspect has been addressed in King Saud bin Abdulaziz University for Health Sciences, which affected the choice of 28% of participants. Patino et al. showed that vision can cause clinically substantial changes in vision-specific health-related standard of living, implying that the ophthalmologist can help patients' lives indirectly [14].

Students have acknowledged the value of lifestyle over time and have made it an intrinsic element of their decision-making factors [4,6,7]. Ophthalmology is one of the recognized specialties that have a satisfying lifestyle that attracts medical students toward it. A satisfying lifestyle is mostly recognized as having sufficient free time off work, which gives the ability to have more time spent with family members, friends, or hobbies. Our study showed that ophthalmology drew 77% of participants because of the satisfying lifestyle and flexible work schedule. Ophthalmology drew 74% of students because of the little contact with emergencies and small range of medical problems. 

Comparing this study with a study done in King Saud bin Abdulaziz University for Health Sciences showed that a lesser degree of stress attracted 24% of their participants toward ophthalmology [11]. Our study showed that 75% were drawn to ophthalmology due to a great selling feature.

The variables that drive people away from a highly competitive specialty like ophthalmology are a fascinating specialty to investigate; we discovered that ophthalmology was avoided by 30% of students due to the chance of having unsatisfied patients and 28% due to the difficulty of getting into the ophthalmology residency program. 

Furthermore, the reliance of ophthalmology on clinical diagnostic skills, the appeal of being an ophthalmologist, and the opportunity to perform procedures were among the aspects that drew our participants in. All of these aspects are supported by independent studies in which students who chose surgical specialties, in general, justified their decision based on the capacity to execute practical procedures and operations, scientific knowledge application, and the prestige of surgery inside the medical field [15,16].

At last, a long-lasting dilemma for medical students remains to be choosing their future career. This may lead to poor decision-making regarding this dilemma. Upon reviewing different studies regarding this issue, we found that most studies do not accommodate a specific specialty, but they approach it in a general manner. Therefore, in our study, we chose to focus on choosing ophthalmology per se as a future career among medical students in different medical schools across all regions of Saudi Arabia. 

## Conclusions

There are many factors that would influence or hold medical students from pursuing ophthalmology as a future career, which would be made clearer with this type of study. Our study is focused on ophthalmology, the factors influencing the choice of ophthalmology, and why it is a competitive field in Saudi Arabia. This research provides a proper insight into what drives students in Saudi Arabia toward and against ophthalmology as future career.
